# HLA class II SNP interactions and the association with type 1 diabetes mellitus in Bengali speaking patients of Eastern India

**DOI:** 10.1186/1423-0127-20-12

**Published:** 2013-02-27

**Authors:** Oindrila Raha, Biswanath Sarkar, Bhaskar VKS Lakkakula, Veerraju Pasumarthy, Sudhakar Godi, Subhankar Chowdhury, Pradip Raychaudhuri, Raghavendra Rao Vadlamudi

**Affiliations:** 1 Anthropological Survey of India, Kolkata, West Bengal 700016, India; 2 Department of Biomedical Sciences, Sri Ramachandra University, Chennai 600 116, India; 3 Department of Human Genetics, Andhra University, Visakhapatnam 530003, India; 4 Endocrinology Department, SSKM Hospital, Kolkata, India; 5 Calcutta Medical College & Hospital, Kolkata, India; 6 Department of Anthropology, University of Delhi, North Campus, Delhi 110 007, India

**Keywords:** HLA, T1DM, SNP, India, Haplotyping, Alleles

## Abstract

**Background:**

Several studies have demonstrated a fundamental role for the HLA in the susceptibility of, or protection to, type 1 diabetes mellitus (T1DM). However, this has not been adequately studied in Asian Indian populations. To assess the frequency of HLA class II (DPA1, DPB1, DQA1, DQB1 and DRB1) associated to susceptibility or protection toT1DM in a Bengali population of India with diabetes.

**Results:**

Single nucleotide polymorphism study. The HLA genotyping was performed by a polymerase chain reaction followed by their HLA-DP, DQ, and DRB1 genotypes and haplotypes by sequencing method. The results are studied by Plink software. The *χ*^2^ tests were used for the inferential statistics. To our knowledge, this study is the first of a kind which has attempted to check the HLA association with T1DM by SNPs analysis. The study recruited 151 patients with T1DM and same number of ethno-linguistic, sex matched non-diabetic controls. The present study found a significant SNP rs7990 of HLA-DQA1 (p = 0.009) negative correlation, again indicating that risk from HLA is considerably more with T1DM.

**Conclusions:**

This study demonstrates that the HLA class-II alleles play a major role in genetic basis of T1DM.

## Background

Type 1 diabetes mellitus (T1DM) (OMIM-222100), results from a cellular-mediated autoimmune destruction of the Beta-cell of the pancreas [1]. T1DM is a disease of major public health concern [2-4]. The previous studies showed that in India, the prevalence of T1DM varies from about 1.6 to 10.5/100000/year [5,6]. The epidemiological study conducted in South Indian population for four years, suggested the prevalence of T1DM in India is increasing. The overall prevalence of T1DM in Karnal district, a North Indian city with a population of 222017, is 10.20/100,000 population [7].

The genetic risk factors of T1DM are better understood than the environmental risk factors. Studies on Human and animal models show the MHC class II–mediated effects on the disease susceptibility [8]. Early studies identified in the Human Leukocyte Antigen (HLA) genes, located on chromosome 6p21.31 as T1DM susceptibility genes. Resulting studies showed an association between the insulin gene on chromosome 11p15.5. The risk of T1DM is linked with about 18 regions of the genome. These regions, each of which may contain multiple genes, labeled IDDM1 to IDDM18. The best studied is IDDM1, which contains the HLA genes encode proteins of the immune response [9,10].

HLA is one of the most polymorphic genetic systems in Human genome. IMGT/HLA database have reported 6,275 HLA alleles [11]. Because the HLA class II molecules are polymorphic, they can embrace a wide variety of antigens in their antigen-binding groove and present them to diverse T-lymphocyte antigen receptors, triggering antigen recognition. Several studies have displayed HLA class II alleles, DQ and DR influence T1DM susceptibility. The contribution of the DQ molecules to overall disease susceptibility might be genotype dependent and/or may be influenced by the DRB1*04 allele on the haplotype [12].

The HLA-DQ heterodimers encoded by the DQA1*0301, DQB1*0302 and DQA1*0501, DQB1*0201 alleles have the strongest association with T1DM [13]. These alleles are in LD with the HLA-DR4 and -DR3 alleles. In T1DM at least one allele of DR3 or DR4 is found in 95% in Europeans, and individuals with both DR3 and DR4 are particularly susceptible to T1DM, whereas, the DR2 allele is protective [14-16].

In North Indian T1DM patients also, the homozygosity and heterozygosity of DRB1*0301 and DRB1*04 alleles is significantly associated [17-19]. The Indian samples show HLA-C*0702 allele and shared HLA-B*0801 and DQB1*02 with the European 8.1AH [20-23].

The role of DP molecules has yet to be resolved satisfactorily. The results favor DPB1*0301 and DPB1*0202 alleles as predisposing for T1DM [24]. Analysis of family-based data from the Human Biological Data Interchange (HBDI) repository and Italian studies, suggests the presence of a T1DM protective locus at or near DPB1*0101 [25]. It is hypothesized that the strongest candidates for increasing T1DM risk among DR3-DQB1*0201/DR4-DQB1*0302 individuals is of alleles of DP and DRB1*04 subtypes and, in particular, the absence of reportedly protective alleles DPB1*0402 and/or DRB1*0403 [26].

There are some studies on HLA-DP in different ethnic groups mainly about HLA-DPB1. In a study from Sudan, there were no significant differences between Sudanese patient and control groups in HLA-DPB1 frequencies [27]. Although, there was also no noticeable association between T1DM and HLA-DPB1 allele in Japanese [28]. From Indian T1DM patients, so far no studies have been reported on DP molecules.

Although different methods exist to characterize the polymorphisms in HLA genes, the 12^th^ International Histocompatibility Workshop suggested the Sequence Based Typing (SBT) methods [29]. Therefore, we conducted a hospital-based case–control study in the West Bengal region of India to find out the role of HLA- DRB1, DQ and DP gene polymorphisms in progress T1DM.

## Methods

### Subjects

In the present study 151 T1DM patients were recruited from six different hospitals - Calcutta Heart and Research Clinic; Endocrinology Department, Calcutta Medical College & Hospital; Endocrinology Department, SSKM Hospital; Netaji Subhash Chandra Bose Cancer Research Institute; Rabindranath Research Institute of Cardiac Sciences; School of Tropical Medicine; from metropolitan Kolkata. The inclusion criteria considered for recruitment of cases were an onset of diabetes below 39 years of age, and presenting with or without acute ketosis with absolute insulin dependence, as shown by a deficient C-peptide secretion i.e., a C-peptide value less than 0.6 (0.6-3.2)ng/ml and antibody positivity for Glutamic Acid Decarboxylase antigen(GADA) and, Insulinoma-Associated Protein-2 Antibodies (/IA-2/ICA512) [30]. Patients of at least one year duration were selected to exclude acute or “honeymoon” phases.

The sampled subjects speak Bengali. The samples represented in our study is mainly originated from districts-Kolkata, followed by South-24-Parganas, North24-Parganas, Howrah, Hoogly, comprising a small geographical area and historically forming a cultural zone. West Bengal is the melting pot of Indo-Aryan, Austric, Dravidian, Tibeto-Burman and various other languages [31]. Some scholar stated that Bengal had many striking resemblance with the Dravidian culture [32], where as others suggested that Bengal as the meeting place of Aryan, non-Aryan, and Mongoloid races [33].

The controls used in the present study represent 151 healthy individuals without T1DM and T2DM in the family history, and matched for ethnicity, geography and socioeconomic status and higher age compared to cases. Blood samples (3 ml) were collected into EDTA-coated vacutainers from both cases and controls with written informed consent. This research was approved by the Institutional Review Board of the Anthropological Survey of India as well as by the respective hospital’s ethical committee.

### Genotyping

DNA was isolated according to the standard protocol [34]. Genotyping of the following HLA genes HLA-DPA1, HLA-DPB1, HLA-DQA1, HLA-DQB1, and HLA-DRB1 was performed using PCR followed by sequencing. The primers used in the present study to amplify different regions of aforementioned genes are documented in Table 1. A total volume of 10 μl was used for each PCR reaction which were carried out in an ABI Gene Amp PCR system 9700. The nucleotide sequences of the PCR products were determined by direct sequencing using di-deoxy chain terminator cycle sequencing protocol through 3730 DNA Analyzer (BigDye V3.1, Applied Biosystems; Foster City, CA, USA). Sequencing was carried out with both the forward and the reverse directions. Problems in genotype assignment for certain samples on the DRB1 and the DQB1 loci that aroused because of the genotypic ambiguity were surmounted by following the guidelines of the American Society for Histocompatibility and Immunogenetics [35].

**Table 1:** List of primers used in the present study to amplify genes

**Designation**	**Chromosome location**	**Sequence**	**Annealing temperature**	**Product size(bp)**
HLA-DPA1*	6p21.3 (Exon2)	F-5’ GCGGACCATGTGTGTCAACTTAT 3’	55°C	210
		R-5’ GCCTGAGTGTGGTTGGAACG 3’		
HLA-DPB1*	6p21.3 (Exon2)	F-5’ GAGAGTGGCGCCTCCGCTCAT 3’	63°C	327
		R-5’ GCCGGCCCAAAGCCCTCACTC 3’		
HLA-DQA1	6p21.3^$^ (Exon1)	F-5’ CAAACTCTTCAGCTAGTAAC 3’	58°C	262
		R-5’ CATGCACTCACCCACAAT 3’		
	6p21.3* (Exon2)	F-5’ ATGGTGTAAACTTGTACCAGT 3’	55°C	229
		R-5’ TTGGTAGCAGCGGTAGAGTTG 3’		
	6p21.3^$^ (Exon3)	F-5’ AGGTTCCTGAGGTCACAGTGTTT 3’	54°C	328
		R-5’ CTTGACAGACAAGAAAGCATC 3’		
HLA-DQB1*	6p21.3(Exon2)	F-5’ CATGTGCTACTTCACCAACGG 3’	58°C	211
		R-5’ CTGGTAGTTGTGTCTGCACAC 3’		
HLA-DRB1*	6p21.3(Exon2)	F-5’ CCCCACAGCACGTTTCTTG 3’	60°C	274
		R-5’ CCGCTGCACTGTGAAGCTCT 3’		

*(Tanaka et al. 1999), ^$^(Cordovado et al. 2005).

### Statistical analysis

All nucleotide changes detected by using SeqScape software v 2.5 (Applied Biosystems) and with the wild gene using pair-wise BLAST [36]. Class II HLA genes are more polymorphic compared to other genes and thus the editing was much more complicated for Class II HLA. Allele and genotype frequencies of the SNP data compared between T1DM patients and healthy control groups. Statistical analysis for HWE was performed by Plink v 1.07 software [37]. Evaluation of the genotype or allele frequencies of cases and controls was carried out by calculating the odds ratios (OR) with 95% of confidence intervals (CI). A *P value* less than 0.05 were considered as statistically significant. Haplotype frequencies and linkage disequilibrium estimated by using Haploview v 4.1, which measures D’ and r^2^ between each pair of SNPs and to define haploblocks [38].

## Results and discussions

**Figure 1:**
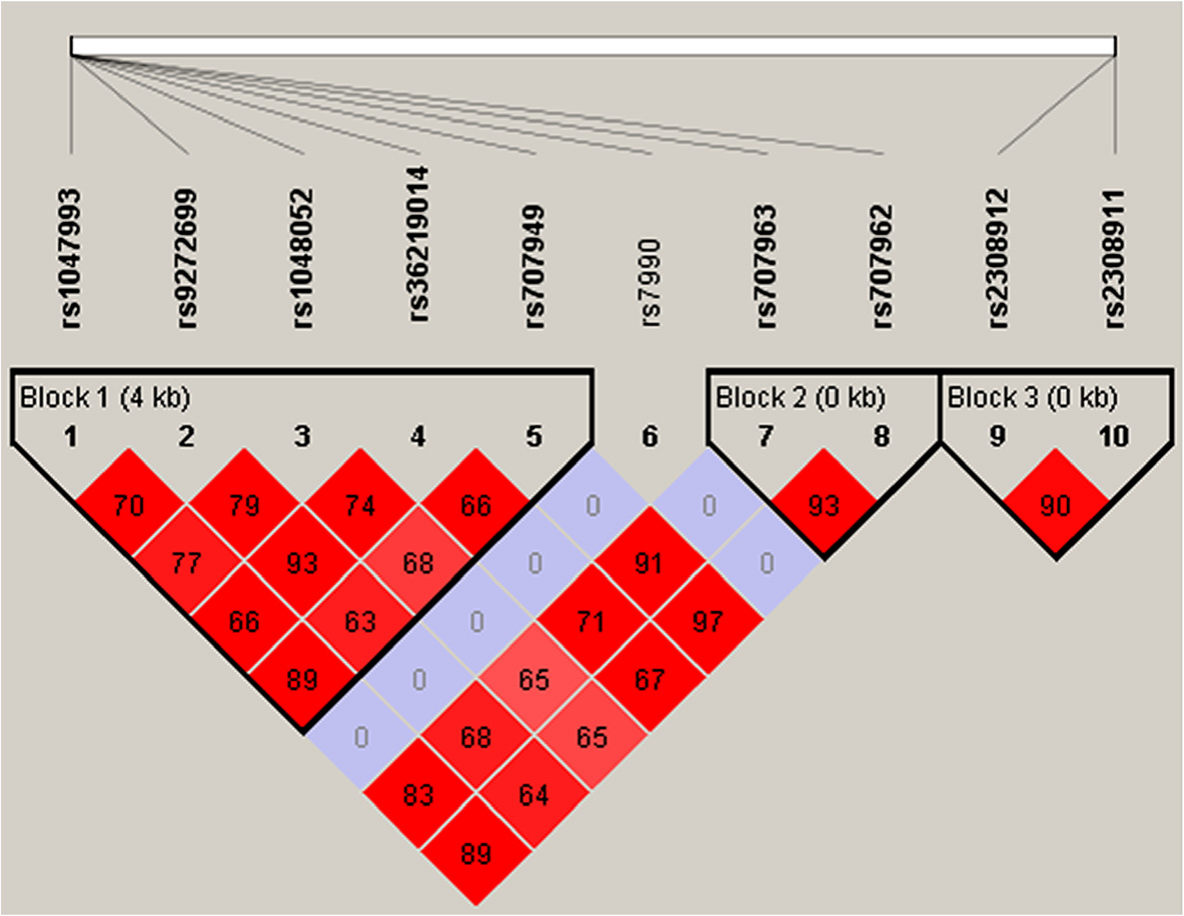
Pair-wise linkage disequilibrium between the ten SNP markers in HLA-DQA1-DPA1 genes.

Exonic 331 SNPs in the Class II HLA- DP-DQ-DRB1, identified and tested for HWE by Plink software. Only ten SNPs found to be in equilibrium for control, only these 10 SNPs were used in further analysis. Of the 10 SNPs, eight were from HLA-DQA1 and the remaining were from HLA-DPA1 (rs2308911, rs2308912) (Table 2). Allele and genotype frequencies of all the SNPs for both cases and controls were presented in Table 2. Except for rs7990 (p = 0.009), there were no significant differences in genotype or allele frequencies of these SNPs between controls and T1DM cases (Table 2). Thus, after all prune and excluding from 331 SNPs, rs7990 SNPs of HLA-DQA1 shows a negative association with T1DM. The pairwise LD values (D’ and r2) among studied SNPs were provided in Figure 1 and in Table 3. Linkage disequilibrium analysis had revealed strong LD and formed 3 haplotype blocks, suggested that haplotype study might be useful. Haplotype-phenotype association analysis using SNPs that were located in the LD blocks in block 1 (rs1047993 and rs707949) showed association with T1DM (Table 3).

**Table 2:** Genotyping of SNPs in LD blocks

	**Genotype**			**MAF (%)**	**HWE-p value**	**Dominant OR (p value)**	**Recessive OR (p value)**	**Allelic OR(p value)**
rs1047993	TT	TC	CC					
Cases	2 (1.45)	26 (18.84)	110 (79.79)	10.9	0.745			
Control	2 (1.33)	18 (12.0)	130 (86.67)	7.3	0.152	1.65 (0.114)	1.09 (0.933)	1.54 (0.139)
rs9272699	AA	AC	CC					
Cases	2 (1.42)	15 (10.64)	124 (87.94)	6.7	0.068			
Control	0 (0)	15 (10.14)	133 (89.86)	5.1	0.516	1.22 (0.603)		1.35 (0.394)
rs1048052	CC	AC	AA					
Cases	4 (2.84)	17 (12.06)	120 (85.11)	8.9	0.003			
Control	0 (0)	17 (11.49)	131 (88.51)	5.7	0.459	1.35 (0.392)		1.60 (0.148)
rs36219014	TT	CT	CC					
Cases	0 (0)	17 (12.06)	124 (87.94)	6	0.446			
Control	0 (0)	15 (10.27)	131 (89.73)	5.1	0.513	120 (0.631)		1.18 (0.642)
rs707962	GG	GT	TT					
Cases	0 (0)	30 (20.69)	115 (79.31)	10.3	0.165			
Control	0 (0)	19 (12.58)	132 (87.42)	6.3	0.409	1.81 (0.061)		1.72 (0.074)
rs2308911	CC	CA	AA					
Cases	15 (10.95)	66 (48.18)	56 (40.88)	35	0.495			
Control	14 (10.37)	61 (45.19)	60 (44.44)	33	0.795	1.16 (0.552)	1.06 (0.877)	1.10 (0.610)
rs2308912	AA	AT	TT					
Cases	14 (10.0)	64 (45.71)	62 (44.29)	32.9	0.669			
Control	12 (9.09)	61 (46.21)	59 (44.70)	32.2	0.502	1.02 (0.946)	1.11 (0.799)	1.03 (0.870)
rs707949	CC	CT	TT					
Cases	0 (0)	30 (20.69)	115 (79.31)	10.3	0.165			
Control	0 (0)	20 (13.25)	131 (86.75)	6.6	0.384	1.71 (0.087)		1.63 (0.104)
**rs7990**	**AA**	**AC**	**CC**					
**Cases**	**0 (0)**	**14 (9.72)**	**130 (90.28)**	**4.9**	**0.549**			
**Control**	**0 (0)**	**31 (20.53)**	**120 (79.47)**	**10.3**	**0.160**	**0.42 (0.009)**		**0.45 (0.013)**
rs707963	GG	GT	TT					
Cases	0 (0)	28 (19.31)	117 (80.69)	9.7	0.198			
Control	0 (0)	18 (11.92)	133 (88.08)	6	0.436	1.77 (0.079)		1.69 (0.093)

MAF = Minor Allele Frequency, HWE = Hardy-Weinberg Equilibrium.

**Table 3:** HLA-DQA1 and-DPA1 gene haplotypes and T1DM

**rs1047993, rs9272699, rs1048052, rs36219014 and rs707949**				
**Haplotype**	**Control**	**Case**	***χ***^**2**^	***p-value***
**CCACT**	**0.883**	**0.939**	**5.672**	**0.017**
TACTC	0.060	0.051	0.255	0.614
**TCACC**	**0.028**	**0.003**	**5.875**	**0.015**
rs707963 and rs707962				
Haplotype	Control	Case	*χ*^2^	*p-value*
TT	0.897	0.937	3.202	0.074
GG	0.097	0.060	2.818	0.093
rs2308911 and rs2308912				
Haplotype	Control	Case	*χ*^2^	*p-value*
TA	0.650	0.663	0.107	0.744
AC	0.336	0.307	0.501	0.479
TC	0.015	0.022	0.436	0.509

**χ **^2^=its chi square.

## Conclusions

The complex nature of the HLA region on chromosome 6p21.31, with the high LD between genes, has made it enormously difficult to explicate the effect of individual genes for the risk of developing T1DM. The studies suggested that HLA Class II DRB1-DQB1 contribute to T1DM susceptibility. Calculating for the influence of class II DR-DQ haplotype and genotype effects, a role in T1DM has been shown of additional HLA Class II DPB1[16].

New studies by sequencing has yielded many SNPs that include thirteen SNPs from class III alleles, which showed evidence of an effect on T1DM risk, although some of the SNPs are in tight LD with each other. The strongest association within class III markers was with rs2395106 that maps 5^′^ to the NOTCH4 gene and the second association was with rs707915 mapping to the MSH5 gene, in a block of six markers significantly associated with T1DM after adjusting for LD with DR-DQ [39].

A widespread SNP analysis of the extended MHC in 237 families with Type 1A from the U.S. and 1,240 families from the T1DGC was conducted and showed an association with Type 1A diabetes (rs1233478, p = 1.6 x 10^-23^), in the UBD/MAS1L region, telomeric of the classic MHC [40]. Another study from T1DGC on HLA markers showed 296 significant SNPs in a narrow genomic region, some of these markers are close to one another and in strong LD. Therefore, although the SNPs that stand for independent signals without LD, some high-LD markers can produce correlated associations in the study. However, high-LD and long haplotype blocks will also deter fine mapping precisely. This study also shows that SNPs with the smallest *P value*s is from the HLA-DR and -DQ region which confers the major genetic risks for T1DM [41].

Most of these recent studies have used data produced by the T1DGC and therefore are from Caucasoid population, and they conducted the most detailed investigation of the HLA complex in disease, characterizing over 3,000 SNPs, and independently tested all previously reported T1DM susceptibility genes [42,43].

In this study we used a sequencing SNP approach to characterize the polymorphisms in HLA genes. We carried out sequencing in 151 cases and 151 normal healthy participants, followed by statistical analysis of the SNPs. Out of 331 exonic SNPs identified, only 10 is following HWE. Thus, after all pruning and excluding from 331 SNPs, and rs7990 SNP of HLA-DQA1 showed significant protection from T1DM. Linkage disequilibrium analysis revealed 3 haplotype blocks and further haplotype-phenotype association analysis did show association between haplotypes in block 1 (rs1047993 and rs707949) and T1DM.

We have also done the study based on alleles and the findings are similar like the DQA1*0103 allele is a novel allele with a significant association with the protection from T1DM in Eastern Indian Bengali population from India [44]. To our knowledge, this study is the first of a kind which has tried to check the HLA association with T1DM by SNPs analysis in India. As said before most of these recent studies have used data formed by the T1DGC, and they use refined statistical methods to control for the complexity of the HLA region because of the extended LD and polymorphic loci. Still the deviation of some results suggests the difficulty of examine the independent genetic contribution of genes in this region to the risk of developing T1DM. It is most likely that more SNPs with individual but smaller or rarer effects on diabetes risk can be identified in this region. However, to find these SNPs new approaches for analyzing genetic association data are needed. In addition, large numbers of subjects may help to give more robust and confident association with T1DM.

## Competing interest

There are no conflicts of interest.

## Authors’ contributions

OR carried out the molecular genetic studies, participated in the sequence alignment, did the statistical calculation and drafted the manuscript. BS corrected the manuscript. BLVKS conducted the Thesia calculation. VP suggested the project GS suggested the project SC provided with the samples PR provided with the samples VRR guided the total project and corrected the MS. All authors read and approved the final manuscript.
